# Sensitivity to radiation-induced chromosome damage may be a marker of genetic predisposition in young head and neck cancer patients

**DOI:** 10.1054/bjoc.2000.1692

**Published:** 2001-03

**Authors:** R Papworth, N Slevin, S A Roberts, D Scott

**Affiliations:** Departments of Cancer Genetics; Department of Clinical Oncology, Christie Hospital NHS Trust, Withington, Manchester, M20 4BX, UK; Biostatistics Paterson Institute for Cancer Research

**Keywords:** head and neck cancer, genetic predisposition, chromosome aberrations, ionizing radiation, lymphocytes

## Abstract

We previously showed that levels of chromosome damage induced by ionizing radiation were, on average, higher in G _2_ and G _0_ lymphocytes of breast cancer patients than of normal healthy controls, but that there was no correlation between the results in the two assays. We proposed that enhanced sensitivity to G _2_ or G _0_ irradiation was a marker of low-penetrance predisposition to breast cancer, and have recently demonstrated heritability of sensitivity in families of breast cancer cases. We have now applied these assays to patients with head and neck cancers, for whom there is epidemiological evidence of inherited predisposition in addition to environmental causes. The mean frequency of radiation-induced G _2_ aberrations was higher in the 42 patients than in 27 normal controls, but not significantly so. However, cases less than 45 years old were significantly more sensitive than normals of the same age range (*P* = 0.046), whereas there was no difference between patients and normals of less than 45 years. Also, there was an inverse correlation between G _2_ sensitivity and age for patients but not for normals. Radiation-induced micronuclei in G _0_ cells were more frequent in 49 patients than in 31 normals (*P* = 0.056) but, as with the G _2_ assay, the greatest difference was seen between early-onset patients and young normals. Again there was an inverse correlation with age for patients but not for normals. Six patients with enhanced toxicity to radiotherapy were G _2_ tested and four other such patients were G _0_ tested; levels of chromosome damage were not significantly greater than in patients with normal reactions. Both assays were used on 64 individuals (39 patients, 25 normals) and there was no significant correlation between the results. We suggest that a proportion of early-onset head and neck cancer patients are genetically predisposed and that each of the two assays detects a different subset of these cases. © 2001 Cancer Research Campaign http://www.bjcancer.com


*British Journal of Cancer* (2001) **84**(6), 776–782
© 2001 Cancer Research Campaign
doi: 10.1054/ bjoc.2000.1692, available online at http://www.idealibrary.com on IDEAL®

http://www.bjcancer.com

### Sensitivity to radiation-induced chromosome damage may be a marker of genetic predisposition in young head and neck cancer patients

**R Papworth[1], N Slevin[2], SA Roberts[3] and D Scott[1]**

Departments of [1]Cancer Genetics and [3]Biostatistics, Paterson Institute for Cancer Research; [2]Department of Clinical Oncology, Christie Hospital NHS Trust, Withington, Manchester M20 4BX, UK

**Summary** We previously showed that levels of chromosome damage induced by ionizing radiation were, on average, higher in $G_2$ and $G_0$ lymphocytes of breast cancer patients than of normal healthy controls, but that there was no correlation between the results in the two assays. We proposed that enhanced sensitivity to $G_2$ or $G_0$ irradiation was a marker of low-penetrance predisposition to breast cancer, and have recently demonstrated heritability of sensitivity in families of breast cancer cases. We have now applied these assays to patients with head and neck cancers, for whom there is epidemiological evidence of inherited predisposition in addition to environmental causes. The mean frequency of radiation-induced $G_2$ aberrations was higher in the 42 patients than in 27 normal controls, but not significantly so. However, cases less than 45 years old were significantly more sensitive than normals of the same age range ($P$ = 0.046), whereas there was no difference between patients and normals of less than 45 years. Also, there was an inverse correlation between $G_2$ sensitivity and age for patients but not for normals. Radiation-induced micronuclei in $G_0$ cells were more frequent in 49 patients than in 31 normals ($P$ = 0.056) but, as with the $G_2$ assay, the greatest difference was seen between early-onset patients and young normals. Again there was an inverse correlation with age for patients but not for normals. Six patients with enhanced toxicity to radiotherapy were $G_2$ tested and four other such patients were $G_0$ tested; levels of chromosome damage were not significantly greater than in patients with normal reactions. Both assays were used on 64 individuals (39 patients, 25 normals) and there was no significant correlation between the results. We suggest that a proportion of early-onset head and neck cancer patients are genetically predisposed and that each of the two assays detects a different subset of these cases. © 2001 Cancer Research Campaign    http://www.bjcancer.com

**Keywords**: head and neck cancer; genetic predisposition; chromosome aberrations; ionizing radiation; lymphocytes

We have shown that lymphocytes of breast cancer patients are, on average, more sensitive than those of normal healthy controls to the induction of chromosome damage by ionizing radiation. This was true for cells irradiated in either the $G_2$ or $G_0$ phases of the cell cycle (Scott et al, 1994, 1998, 1999). The $G_2$ assay involved the analysis of metaphase cells for structural aberrations whereas, in the $G_0$ assay, chromosome damage was measured as the induction of micronuclei (MN). Our $G_2$ observations have now been confirmed in three independent studies in different laboratories (Parshad et al, 1996; Patel et al, 1997; Terzoudi et al, 2000).

Using the $G_2$ assay on 105 normal individuals we found a skewed distribution of induced aberration yields, with 5–10% of donors being sensitive outliers. This proportion was much higher (42%) among 135 breast cancer patients (Scott et al, 1999). With the MN assay we found that 27% (35 of 130) of patients were of elevated sensitivity, compared with 10% (7 of 68) of normals. When we performed both assays on the same 80 patients we found no evidence of a correlation between aberration yields in the $G_2$ assay and MN yields in the $G_0$ assay (Scott et al, 1999) suggesting that the cellular defects leading to enhanced sensitivity are different in these cell cycle stages.

*Received 9 October 2000*
*Revised 11 December 2000*
*Accepted 19 December 2000*

*Correspondence to:* SA Roberts; E mail: sroberts@picr.man.ac.uk

We have recently shown that the degree of sensitivity in the $G_2$ assay is an inherited characteristic in the families of patients with breast cancer and could be attributed to the segregation of one or two genes in each family (Roberts et al, 1999; Scott et al, 2000). We also have preliminary evidence that elevated sensitivity in the $G_0$/MN assay is a heritable trait in first-degree relatives of breast cancer patients (Burrill et al, 2000).

These observations in breast cancer patients and their families have led us to suggest that such enhanced chromosomal radiosensitivity may be a marker of cancer-predisposing genes. Support for this hypothesis comes from the demonstration that many inherited cancer-prone conditions (e.g. ataxia-telangiectasia, Li–Fraumeni syndrome, hereditary retinoblastoma) exhibit evidence of this type of elevated radiosensitivity (reviewed in Scott et al, 1999) but, in contrast to the situation in our breast cancer studies, the gene defects responsible for cancer predisposition in these rare syndromes are generally strongly expressed (highly penetrant). We propose that the defects leading to the enhanced radiosensitivity that we have seen in our studies are associated with a lesser risk of cancer and therefore do not lead to a strong family history (low-penetrance genes). There is good epidemiological evidence that the inherited risk of breast cancer is greater than can be accounted for by mutations in the highly penetrant genes *BRCA1*, *BRCA2* and *TP53* (Teare et al, 1994; Lichtenstein et al, 2000; Peto and Mack, 2000).

There is also indirect evidence for the existence of low-penetrance, inherited, predisposing factors for cancers other than breast; for example, lung (Sellers, 1996), colorectal (Cannon-Albright et al, 1988) and head and neck cancers. For the latter group, Foulkes et al (1995) found, in a case-control study, that even when allowing for the known environmental risk-factors such as alcohol and tobacco consumption, cancer in a first-degree relative was a significant independent risk-factor.

In the present study, we have investigated the chromosomal radiosensitivity of head and neck cancer patients and normal healthy controls, using both the $G_2$ and $G_0$ assays. Because it has been suggested that genetic factors may be particularly important in young patients with head and neck cancers, where there will be a reduced impact of cumulative environmental factors (Son and Kapp, 1985), our selection of cancer cases has been biased in favour of such early-onset patients. Our sample of patients also included a small number of cases who had shown adverse reactions to radiotherapy, because we have previously shown that the average radiosensitivity of *breast* cancer patients of this type may be greater than that of normally-reacting patients, depending upon the nature of the reactions and the type of assay (Barber et al, 2000).

### MATERIALS AND METHODS

### Patients and normal controls

Individuals tested with the $G_2$ and/or the $G_0$ assay comprised 4 groups:

1. Healthy subjects (normals), mainly from within the staff of this Institute but including a small number of spouses of patients
2. Head and neck cancer patients at the Christie Hospital before they received radiotherapy (pre-therapy cases)
3. Patients after radiotherapy (9 months to 10 years post-therapy, mean 5.7, SD 2.5 years). These will be referred to as post-therapy cases
4. A small group of patients after radiotherapy (2–5 years, mean 3.7, SD 1.2) for whom the treating clinician identified radiation necrosis as a late complication following a standard radiotherapy schedule. These are designated ‘highly radiosensitive’ (HR) patients according to the nomenclature of Burnet et al (1998).

The majority (36 of 50) of the patients had tumours of the larynx, other sites being mouth, tongue, tonsil, oral cavity and oropharynx. The distribution of sites was not significantly different between patients groups 2–4. Over the period of this study the proportion of early onset (<45 years) laryngeal cancer cases admitted to this hospital was 3.3%, whereas in our sample the proportion was 21% (11 of 52), indicating our preferential selection of younger cases.

Details of tobacco and alcohol consumption were obtained from those patients who volunteered this information, but not from normals. Table 1 shows the characteristics of the various patient groups. Permission for the study was obtained from the local Ethics Committee.

### The $G_2$ assay

Full details are given in Scott et al (1999). Briefly, whole-blood cultures were set up in pre-warmed (37˚C) and pre-gassed (5% $CO_2$, 95% air) medium. One hour later, lymphocytes were stimulated with phytohaemagglutinin (PHA) and cultured for 70 h, at which time the culture medium was replaced, without centrifugation, with fresh medium. Cells were irradiated (or mock-irradiated) at 72 h with 0.5 Gy 300 Kv X-rays, colcemid was added 30 min later and at 90 min after irradiation culture vessels were plunged into ice chippings. Subsequent centrifugation, hypotonic treatment and fixation was carried out at 4˚C. From 1 h before irradiation to the time of harvesting, cultures were kept at 37˚C.

Metaphase preparations were made with standard procedures and Giemsa stained. Slides were randomized and coded for analysis and 50–100 metaphases were scored from both irradiated and control samples. The frequency of aberrations in control samples was subtracted from that in irradiated samples to give the induced yield. The majority of aberrations were chromatid breaks which were misaligned with respect to the intact sister chromatid or, if aligned, had an achromatic region of greater than the width of the chromatid. Smaller achromatic lesions (gaps) and occasional radiotherapy-induced chromosome-type aberrations in patients were ignored.

### The $G_0$ micronucleus assay

These experiments were performed before we had standardized our MN assay (Scott et al, 1999) so the procedures differ in several respects from those used in our studies of breast cancer patients.

Heparinized whole blood was kept overnight (16–24 h) at room temperature, then 0.5 ml aliquots were added to 4.5 ml of culture medium which comprised 82% RPMI 1640 (Flow Laboratories, Ashby de la Zouche, UK), 15% fetal calf serum (FCS) (Gibco BRL, Lewes, UK), 1% L-glutamine (Gibco BRL) and 2% of a mixture of penicillin and steptomycin (both at 5000 units $ml^{-1}$). The medium was in T-25 flasks (Corning Costar, High Wycome, UK) and was pre-warmed (37˚C) and pre-gassed (5% $CO_2$, 95%

**Table 1** Details of participants

| Series | Group | n | M/F | Mean age at assay (SD) | Mean age at diagnosis (SD) | Tobacco: mean pack-years (SD) | Alcohol: mean units per week (SD) |
|---|---|---|---|---|---|---|---|
| $G_2$ | Normals | 27 | 10/17 | 48.6 (17.2) | – | – | – |
| | Pre-therapy | 16 | 12/4 | 60.3 (12.2) | * | 24.8 (21.0) $n$ = 10 | 16.2 (12.4) $n$ = 9 |
| | Post-therapy | 20 | 16/4 | 52.2 (13.6) | 46.6 (11.6) | 17.8 (18.0) $n$ = 18 | 23.4 (29.6) $n$ = 10 |
| | HR | 6 | 4/2 | 62.7 (5.1) | 59.3 (6.3) | 20.7 (13.3) $n$ = 3 | 34.0 (31.1) $n$ = 2 |
| $G_0$ | Normals | 31 | 14/17 | 48.5 (17.0) | – | – | – |
| | Pre-therapy | 22 | 16/6 | 61.0 (13.3) | * | 22.2 (18.2) $n$ = 14 | 15.7 (11.2) $n$ = 13 |
| | Post-therapy | 23 | 18/5 | 54.7 (14.7) | 49.0 (12.7) | 20.1 (18.8) $n$ = 21 | 27.8 (28.7) $n$ = 14 |
| | HR | 4 | 2/2 | 62.0 (4.2) | 58.5 (5.4) | 28.0 (5.7) $n$ = 2 | 12.0 $n$ = 1 |

* Pre-therapy cases assayed shortly after diagnosis

© 2001 Cancer Research Campaign air). One hour after setting up the cultures they were irradiated (or mock-irradiated) with 3.0 Gy $^{137}$Cs gamma rays at 3.3 Gy min$^{-1}$ and returned to the incubator for 1 h, at which time PHA was added at a final concentration of 1.0 μg ml$^{-1}$. At 24 h after PHA stimulation, 3 ml of culture medium was pipetted from each culture flask and replaced with fresh, pre-warmed and pre-gassed medium, then cytochalasin-B was added at a final concentration of 6 μg ml$^{-1}$ to enable the identification of post-mitotic cells as binucleates (Fenech and Morley, 1985).

At 72 h after stimulation, 'clean' cytospin preparations were made, first by separating the lymphocytes from other cells (mainly erythrocytes) in the culture medium by layering the contents of each flask onto 5 ml of Lymphoprep (Nycomed, Amersham, UK) in a 12.5 ml centrifuge tube and centrifuging at 1100 rpm for 30 min. Then, an aliquot of the lymphocyte-rich buffy coat was removed with a small pipette, suspended in 5 ml of PBS and centrifuged at 1500 rpm for 5 min. The latter procedure was repeated and cells were resuspended in 1 ml of PBS. Aliquots of 100–200 μl were then pipetted into cytofunnel chambers and spun onto clean microscope slides by cytocentrifugation for 2 min at 1000 rpm. Cells were fixed in 90% methanol, dried, stained with 10% Giemsa for 10 min, rinsed in distilled water, dried and mounted.

Slides were randomized and coded and a minimum of 100 binucleate cells was scored for MN from both irradiated and control samples.

The principal differences between this protocol and that which has now become our standard procedure (Scott et al, 1999) are: a radiation dose of 3 Gy (3.5 Gy in our standard assay), a delay of 1 h between irradiation and addition of PHA (cf. 6 h), fixation at 72 h after stimulation (cf. 90 h) and cell preparation by cytocentrifugation (cf. conventional harvesting with a short hypotonic treatment). Cells were scored using similar criteria for both assays but by different microscopists.

### Statistical methods

Assay variability was assessed using standard one-way analysis of variance. Aberration yields were compared using Mann–Whitney U-tests, supplemented with Kruskall–Wallis tests where there were more than two groups being compared. Proportions of sensitive cases were compared using Fisher's exact tests. Spearman's rank correlations were used to look at associations between aberration yields and age. A significance level of 0.05 was used throughout.

### RESULTS

A total of 69 individuals were tested with the $G_2$ assay, 80 with the $G_0$ assay (Table 1) and 64 with both (see Figure 5). When both assays were used, this was with the same blood sample.

### The $G_2$ assay

The mean spontaneous yield of aberrations in the various patient groups was slightly, but not significantly, above the level of 1.2 ± 1.5 per 100 cells in normal donors.

To assess assay reproducibility, six normal donors were tested on two (four donors) or three (two donors) occasions. The intra-individual coefficient of variation (CV) for radiation-induced aberration yields, which is a measure of assay error, was 7.3%, very similar to the value of 7.0% which was our previous estimate from repeat assays on 28 normal donors (Scott et al, 1999).

The mean yield of induced aberrations in the 27 normals tested in this study, was 117.7 ± 14.5 per 100 cells (Table 2), which is higher than that from our earlier investigation of 105 normals (97 ± 15, Scott et al, 1999). This is likely to be because the samples from the two studies were scored by different microscopists and probably reflects differences in the inclusion of small gaps in the scores (see above). Although the mean yield in the 42 patients was higher than normals, for none of the three patient subgroups (pre-therapy, post-therapy or highly-radiosensitive) was this increase statistically significant (Table 2, Figure 1). The highest yields were seen in the post-therapy patients (127.0 ± 19.7) but this level was not significantly ($P$ = 0.13) above that in the pre-therapy group (117.3 ± 14.4). There was no indication that the scores for the six highly-radiosensitive (HR) patients were higher than those of the 20 post-therapy cases with normal reactions to radiotherapy.

A method of comparing different groups of individuals, other than simply using mean values, is to chose a cutoff value between a normal and a sensitive response for healthy donors and to compare this proportion of sensitive cases with the proportion of patients whose yields are above the cutoff value (Table 2, Figure 1). Previously, we have chosen the 90th percentile as the cutoff (Scott et al, 1999). Using this criterion, the cutoff value in the present study was 135 aberrations per 100 cells. This actually gave

**Table 2** Yields of induced $G_2$ aberrations or MN, and the proportions of sensitive cases, for normals and for the various subgroups of patients (see also Figures 1 and 3)

| Assay | | Sensitive | | | | Fishers' Exact *P* | | Mean (SD) | Mann–Whitney *P* | |
|---|---|---|---|---|---|---|---|---|---|---|
| | | *n* | sens (*n*) | % | 95% CI | vs Normals | vs Post | | vs Normals | vs Post |
| $G_2$ | Normals | 27 | 4 | 15 | 4–34 | * | – | 117.7 (14.5) | | |
| | All cancer | 42 | 13 | 31 | 18–47 | 0.16 | – | 122.4 (17.9) | 0.46 | – |
| | Pre-therapy | 16 | 2 | 13 | 2–38 | 1.0 | 0.067 | 117.3 (14.4) | 0.77 | 0.13 |
| | Post-therapy | 20 | 9 | 45 | 23–68 | 0.045 | * | 127.0 (19.7) | 0.16 | * |
| | HR | 6 | 2 | 33 | 4–78 | 0.30 | 1.0 | 121.3 (19.4) | 0.80 | 0.74 |
| $G_0$ | Normals | 31 | 3 | 10 | 2–26 | * | – | 50.6 (5.8) | * | – |
| | All cancer | 49 | 17 | 35 | 22–50 | 0.016 | – | 55.5 (10.2) | 0.056 | – |
| | Pre-therapy | 22 | 9 | 41 | 21–64 | 0.017 | 0.35 | 55.8 (11.8) | 0.26 | 0.94 |
| | Post-therapy | 23 | 6 | 26 | 10–48 | 0.15 | * | 54.1 (8.4) | 0.14 | * |
| | HR | 4 | 2 | 50 | 7–93 | 0.089 | 0.56 | 62.0 (9.8) | 0.011 | 0.13 |

*Reference group

 © 2001 Cancer Research Campaign

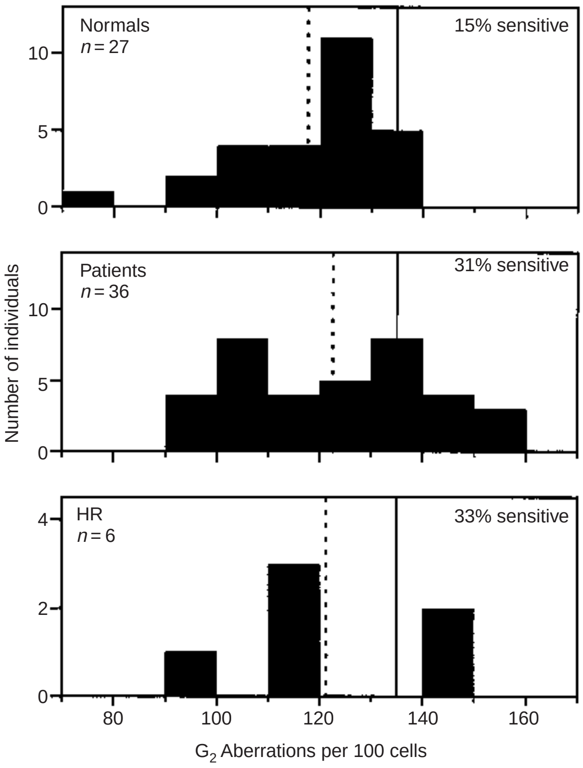

**Figure 1** Radiation-induced $G_2$ aberration yields in normals and in the various subgroups of patients (see also Table 2). The cutoff used to define the sensitive population is indicated by the solid vertical line, and the mean aberration yields of each group are shown as broken vertical lines

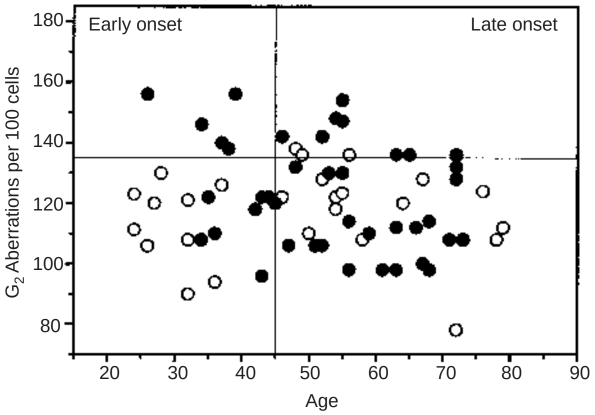

**Figure 2** The relationship between induced $G_2$ aberration yields and age at diagnosis (patients = closed symbols) or at the time of testing (normals = open symbols). See also Table 3. The vertical and horizontal lines indicate the cutoff values used to define sensitivity in the two assays

15% (4 of 27), not 10%, sensitive normals because the $G_2$ score for several individuals fell exactly on the cutoff value. For all 42 patients, the proportion of sensitive cases was 31% (13 of 42) but this was not significantly higher ($P = 0.16$) than the 15% of sensitive normals. Of the various patient subgroups, only the post-therapy group had a sensitive proportion (45%, 9 of 20) that was significantly higher than normals ($P = 0.045$). This proportion of sensitive post-therapy patients was higher than that for pre-therapy cases (13%, 2 of 16), but the difference did not quite reach statistical significance ($P = 0.067$).

There was no indication of any influence of age on radiosensitivity for normal donors ($r = 0.002$, $P = 0.99$, Figure 2), but for patients there was an inverse correlation with age at diagnosis ($r = 0.32$, $P = 0.038$, Figure 2). It should be pointed out that the average age of the patients was greater than that of the normals (Table 1). To further investigate the influence of age on sensitivity in the assay we have stratified the patients into early (≤45 years) and normal (> 45) onset cases. The mean induced $G_2$ yield of early-onset cases (127.2 ± 18.6, Table 3) was greater than that of young (< 45 years) normals (112.9 ± 13.5, $P = 0.12$) and when the difference between patients and normals was expressed in terms of the proportion of sensitive cases, the difference was statistically significant

**Table 3** Yields of induced $G_2$ aberrations or MN in donors who were above or below the age of 45 years at the time of diagnosis (patients) or testing (normals) (see also Figures 2 and 4)

| Assay | | Sensitive | | | Fishers' Exact *P* | | | Mean (SD) | Mann–Whitney *P* | | |
|---|---|---|---|---|---|---|---|---|---|---|---|
| | | *n* | sens (*n*) | % | vs Age-matched normals | vs All normals | Early vs Late | | vs Age-matched normals | vs All normals | Early vs Late |
| $G_2$ | All Normals | 27 | 4 | 15 | | * | – | 117.7 (14.5) | | * | – |
| | Early-onset | | | | | | | | | | |
| | Normals | 10 | 0 | 0 | * | – | * | 112.9 (13.5) | * | – | * |
| | Cancer | 13 | 5 | 38 | 0.046 | 0.12 | * | 127.2 (18.6) | 0.12 | 0.23 | * |
| | Late-onset | | | | | | | | | | |
| | Normal | 17 | 4 | 24 | * | – | 0.26 | 120.4 (14.7) | * | – | 0.14 |
| | Cancer | 29 | 8 | 28 | 1.0 | 0.33 | 0.50 | 120.3 (17.5) | 0.69 | 0.77 | 0.24 |
| $G_0$ | All Normals | 31 | 3 | 10 | | * | – | 50.6 (5.8) | | * | – |
| | Early-onset | | | | | | | | | | |
| | Normals | 11 | 1 | 9 | * | – | * | 50.0 (6.1) | * | – | * |
| | Cancer | 13 | 7 | 54 | 0.033 | 0.003 | * | 59.1 (9.7) | 0.026 | 0.008 | * |
| | Late-onset | | | | | | | | | | |
| | Normal | 20 | 2 | 10 | * | – | 1.0 | 51.0 (5.7) | * | – | 0.82 |
| | Cancer | 36 | 10 | 28 | 0.18 | 0.072 | 0.17 | 54.2 (10.2) | 0.38 | 0.26 | 0.15 |

*Reference groups

*© 2001 Cancer Research Campaign*

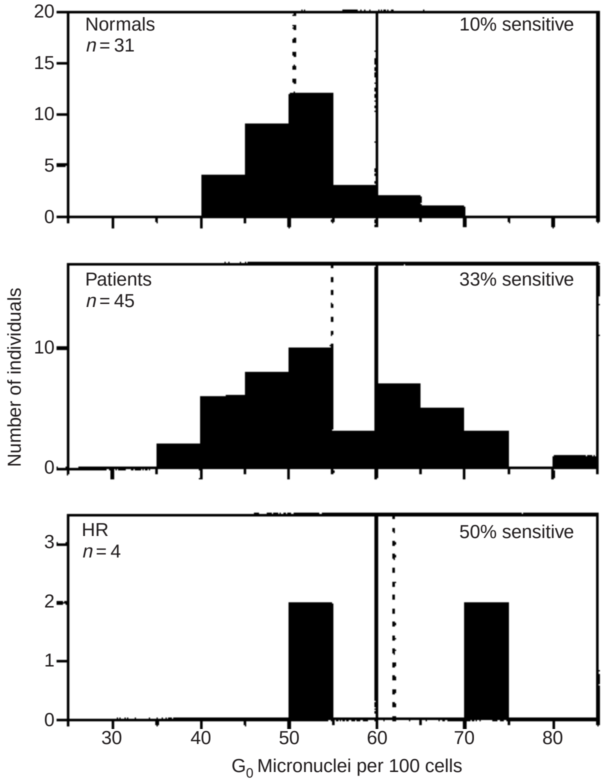

**Figure 3** Radiation-induced MN yields in normals and in the various subgroups of patients (see also Table 2). The cutoff used to define the sensitive population is indicated by the solid vertical line, and the mean aberration yields of each group are shown as broken vertical lines

**Table 4** Smoking and alcohol consumption in early- or normal-onset patients tested with the $G_2$ or $G_0$ assays. Not all patients volunteered this information and the numbers of responses is indicated

| Series | | Smoking Mean pack-years (SD) | Alcohol Mean units per week (SD) |
|---|---|---|---|
| $G_2$ | Early-onset (≤ 45) | 10.4 (9.9) *n* = 12 | 7.8 (9.7) *n* = 4 |
| | Late-onset (> 45) | 26.6 (19.9) *n* = 19 | 24.5 (24.4) *n* = 17 |
| | Mann–Whitney *P* | 0.014 | 0.12 |
| $G_0$ | Early-onset (≤ 45) | 11.3 (11.3) *n* = 12 | 20.2 (29.1) *n* = 5 |
| | Late-onset (> 45) | 26.2 (18.6) *n* = 25 | 21.9 (21.2) *n* = 23 |
| | Mann–Whitney *P* | 0.016 | 0.45 |

(38% sensitive patients, 0% sensitive normals, $P = 0.046$). On the other hand, mean yields and sensitive proportions were very similar for patients and normals above the age of 45 years (Table 3). There was a wide range in smoking and alcohol consumption in both groups, the mean consumption being higher in the older patients, the difference reaching statistical significance for smoking but not for alcohol use (Table 4). There was no significant correlation between the induced $G_2$ yield and smoking or alcohol consumption.

There was no influence of gender on either spontaneous or induced aberration frequencies.

### The MN assay

The spontaneous MN yield in the patients was not significantly different from the level of 3.5 ± 2.6 in normals.

Assay error for induced MN yields, estimated from repeat tests on six normal donors (three tested twice and three tested three times) was 6.2%, less than our previous estimate of 13% from repeat tests on 14 normals (Scott et al, 1999).

The mean yield of induced MN for all 49 patients (55.6 ± 5.8 per 100 cells) was higher than that of the 31 normals (50.6 ± 10.2), on the borderline of significance ($P = 0.056$, Table 2). When the patients were stratified into their various subgroups (Table 2, Figure 3) mean yields were higher than normals but the level of statistical significance was less, because of the relatively small numbers of patients in each subgroup, except for the four HR patients whose mean yield (62.0 ± 9.8) was significantly above the normals ($P = 0.011$). However, the more appropriate group to compare with the HR cases are the post-therapy patients with a normal response to therapy. The yield in HR patients was not significantly higher than that in these normal responders (54.1 ± 8.4). The response of pre- and post-therapy patients was not significantly different. The range of values for patients was greater than that of normals (Figure 3).

Using the 90th percentile of healthy donors to distinguish sensitive from normal responses gave a cutoff value of 60 MN per 100

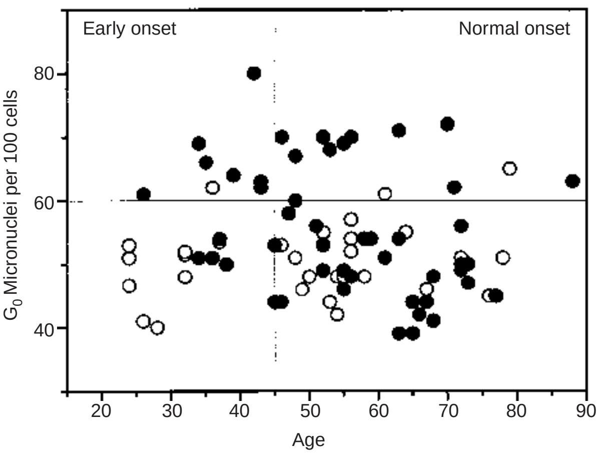

**Figure 4** The relationship between induced MN yields and age at diagnosis (patients = closed symbols) or at the time of testing (normals = open symbols) see also Table 3. The vertical and horizontal lines indicate the cutoff values used to define sensitivity in the two assays

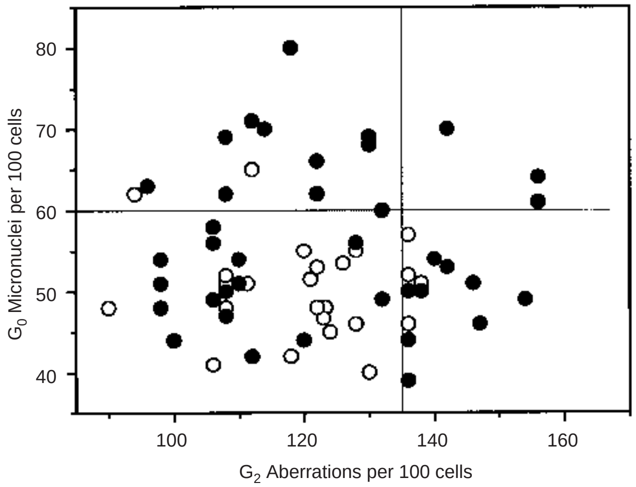

**Figure 5** Radiation-induced MN yields and $G_2$ aberrations for the same 64 donors, using the same blood sample for both assays (see also Table 4). Closed symbols are patients and open symbols are normals. The vertical and horizontal lines indicate the cutoff values between normal and sensitive responses in the $G_0$ and $G_2$ assays respectively

*© 2001 Cancer Research Campaign*

cells (Figure 3). The proportion of all patients above this cutoff value was 35%, which was significantly higher ($P = 0.016$) than the 10% value for normals. Each of the patient subgroups had sensitive proportions above the normals, significantly so for the pre-therapy group ($P = 0.017$).

There was no significant influence of age on the response of normals ($r = 0.22$, $P = 0.23$, Figure 4) but, as with the $G_2$ assay, there was a significant inverse correlation for patients ($r = 0.30$, $P = 0.035$, Figure 4). Again as with the $G_2$ assay, the mean MN yield in patients under 45 years at diagnosis (59.1 ± 9.7) was higher ($P = 0.026$) than that of the normals of < 45 years (50.0 ± 6.1, $P = 0.026$), whereas there was no significant difference between patients and normals of > 45 years ($P = 0.38$, Table 3). Similarly, the proportion of sensitive young patients (54%, 7 of 13) was significantly higher than that of young normals (9%, 1 of 11, $P = 0.003$), whereas the difference in the sensitive proportions of older patients and normals did not reach statistical significance ($P = 0.18$). Smoking and alcohol consumption were, on average, higher in the older patients, significantly so for smoking (Table 4). However, there were no significant correlations between MN yields and smoking or alcohol consumption.

There was no difference in spontaneous or induced yields of MN between males and females.

### Both assays

A total of 64 individuals were tested with both assays on the same blood sample. These comprised 25 normals and 39 patients (16 pre-therapy, 19 post-therapy and four HR cases). There was no significant correlation between the results of the two assays ($r = 0.05$, $P = 0.81$ for normals, $r = 0.40$, $P = 0.13$ for patients, see Figure 5). The proportion of individuals who were sensitive in both assays (5% of those tested, Figure 5) was very close to that predicted if the results of both assays are completely uncorrelated (6%). This was also true for the various subgroups of donors.

### DISCUSSION

We have previously argued that enhanced chromosomal radiosensitivity may be a marker for low-penetrance predisposition to breast cancer. We have now applied both the $G_2$ and $G_0$ micronucleus assays to patients with head and neck cancers for which there is epidemiological evidence of inherited risk in spite of a strong environmental influence, particularly through tobacco and alcohol useage (Morita et al, 1994; Copper et al, 1995; Foulkes et al, 1995).

### The $G_2$ assay

With the $G_2$ assay, although the mean yield of aberrations and the proportion of sensitive cases was higher for all of the patient groups compared with the normals, this increase was not statistically significant (Table 2). However, when patients were stratified on the basis of age of onset of disease, early-onset cases (<45 years) were significantly more sensitive than normals in this age group, whereas later-onset cases (> 45 years) were of very similar sensitivity to normals of corresponding age (Table 3).

Also, there was a significant negative correlation between aberration yields and age for patients but not for normals. If $G_2$ chromosomal radiosensitivity is indicative of genetic predisposition to head and neck cancers, as we have suggested for breast cancer, the above results would indicate that for early-onset cases there is a genetic contribution to risk, but not so for normal-onset cases. For the latter, environmental influences may predominate. It should be noted that smoking and alcohol consumption were higher in the latter group (Table 4). There is some evidence that head and neck cancers in young adults may be clinically different from those in older patients, tending to be more anaplastic and consequently more aggressive (Son and Kapp, 1985) although this difference has not been seen in all studies (Von Doersten et al, 1995).

These results for head and neck cancer patients differ from those for breast cancer cases in that there was no age-dependence for $G_2$ sensitivity in the latter group (Scott et al, 1999). The proportion of young head and neck cases that were sensitive (38%) was similar to that for *all* breast cancer patients (42%), but since early-onset head and neck cancers represent <5% of all cases (references in Son and Kapp, 1985), our results with the $G_2$ assay would suggest a considerably lower genetic component in the overall risk of head and neck cancer than for breast cancer. Terzoudi et al (2000) recently reported that the mean $G_2$ sensitivity of 185 patients with various cancers was significantly higher than that of 25 normals. Among the patients were 20 cases of laryngeal cancer whose $G_2$ scores were higher than those of the normals, although the statistical significance of this increase was not given and the ages of the patients were not specified.

Enhanced sensitivity of $G_2$ lymphocytes of head and neck cancer patients to the chromosome-damaging agent, bleomycin, has been reported in several studies (references in Cloos et al, 1996). In a large case-control study of risk-factors for head and neck cancer, in which age, history of tobacco and alcohol usage, and bleomycin $G_2$ sensitivity were recorded, it was shown that the latter parameter is a biomarker of cancer susceptibility, since it modulates the risk from carcinogen exposure (Cloos et al, 1996). It has also been shown that, as in the case of $G_2$ X-ray sensitivity (Roberts et al, 1999), there is a strong inherited component in $G_2$ bleomycin sensitivity (Cloos et al, 1999). However, $G_2$ response to X-rays cannot simply be regarded as a surrogate for response to bleomycin because, although breast cancer cases show enhanced X-ray sensitivity, they exhibit a normal bleomycin response (Hsu et al, 1989). Also, unlike our present observations on head and neck cancer patients, Cloos et al (1996) found a significant *positive* correlation between age and $G_2$ bleomycin sensitivity in 313 such patients.

The fact that we were unable to distinguish between patients who had shown late HR reactions or normal responses to radiotherapy with the $G_2$ assay agrees with our studies on breast cancer patients, where this assay was only able to distinguish patients with *acute* HR reactions (Barber et al, 2000). In the present study and that on breast cancer patients there was an indication that non-HR patients tested post-therapy were more sensitive than pre-therapy patients, but in neither case was this difference statistically significant. The possibility that radiotherapy may alter the response of lymphocytes in the $G_2$ assay requires further investigation on the same group of patients tested before and after treatment.

### The micronucleus assay

As we found in our studies of breast cancer patients (Scott et al, 1999), in the present investigations we found no significant correlation between the results of the $G_2$ and $G_0$ assays. This suggests that different mechanisms are responsible for enhanced sensitivity in the two tests and that these assays are independent

© 2001 Cancer Research Campaign markers of predisposition to both breast and head and neck cancers.

Using either the mean MN yields or the proportion of sensitive cases, there was better discrimination between patients and normals with this assay than with the $G_2$ assay (Table 2). However, as with the $G_2$ assay, this difference was seen mainly in early-onset patients where 54% were sensitive compared with 9% normals (Table 3). The inverse correlation between MN yields and patient age differs from that for breast cancer patients, where no significant trend was seen (Scott et al, 1999). Further quantitative comparisons with the MN and breast cancer data are probably of limited value because of differences between the assays used in the two studies (Materials and methods).

Rached et al (1998) showed that the average sensitivity of 15 cancer patients was greater than that of 15 normals, using a lymphocyte MN assay. The patients included eight cases of head and neck cancer but their individual MN scores and ages were not given.

There was a suggestion of enhanced mean sensitivity of the four patients who had shown adverse late reactions to radiotherapy, compared with 23 normally-reacting cases, but the difference was not significant. In a study of a larger number of breast cancer patients we obtained better discrimination between severe late reactors and normal reactors but, again, there was a complete overlap of values for the two groups, which obviously limits the value of the assay for predictive purposes (Barber et al, 2000).

Our main finding is that both assays are able to identify chromosomally radiosensitive groups of early-onset patients who may be genetically predisposed to head and neck cancer, each assay detecting a different subgroup of these patients.

### ACKNOWLEDGEMENTS

We wish to thank all the patients and normal donors who participated in this study. Funding of the first author was provided by the Westlakes Research Institute, Cumbria, UK. Additional support was from the Cancer Research Campaign.

### REFERENCES

Barber JPB, Burrill W, Spreadborough AR, Levine E, Warren C, Kiltie AE, Roberts SA and Scott D (2000) Relationship between in vitro chromosomal radiosensitivity of peripheral blood lymphocytes and the expression of normal tissue damage following radiotherapy for breast cancer. *Radiother Oncol* **55**: 179–186

Burnet NG, Johansen J, Turesson I, Nyman J and Peacock JH (1998) Describing patients' normal tissue reactions: concerning the possibility of individualising radiotherapy dose prescriptions based on potential predictive assays of normal tissue radiosensitivity. *Int J Cancer* **79**: 606–613

Burrill W, Barber JBP, Roberts SA, Bulman B and Scott D (2000) Heritability of chromosomal radiosensitivity in breast cancer patients: a pilot study with the lymphocyte micronucleus assay. *Int J Radiat Biol* **76**: 1617–1619

Cannon-Albright LA, Skolnick MH, Bishop T, Lee RG and Burt RW (1988) Common inheritance of susceptibility to colonic adenomatous polyps and associated colorectal cancers. *New Engl J Med* **319**: 533–537

Cloos J, Spitz MR, Schantz SP, Hsu TC, Zhang Z-f, Tobi H, Braakhuis BJM and Snow GB (1996) Genetic susceptibility to head and neck squamous cell carcinoma. *J Natl Cancer Inst* **88**: 530–535

Cloos J, Nieuwenhuis EJC, Boomsma DI, Kuik DJ, van der Sterre MLT, Arwert F, Snow GB and Braakhuis BJM (1999) Inherited susceptibility to bleomycin-induced chromatid breaks in cultured peripheral blood lymphocytes. *J Natl Cancer Inst* **91**: 1125–1130

Copper MP, Jovanovic A, Nauta JP, Braakhuis BJM, de Vries N, van der Waal I and Snow GB (1995) *Arch Otolaryngol Head Neck Surg* **121**: 157–160

Fenech M and Morley AA (1985) Measurement of micronuclei in lymphocytes. *Mutation Res* **147**: 29–36

Foulkes WD, Brunet J-S, Kowalski LP, Narod SA and Franco E (1995) Family history of cancer is a risk factor for squamous cell carcinoma of the head and neck in Brazil: a case-control study. *Int J Cancer* **63**: 769–773

Hsu TC, Johnston DA, Cherry LM, Ramkissoon D, Schantz SP, Jessup JM, Winn RJ, Shirley L and Furlong C (1989) Sensitivity to genotoxic effects of bleomycin in humans: possible relationship to environmental carcinogenesis. *Int J Cancer* **43**: 403–409

Lichtenstein P, Holm NV, Verkasalo PK, Iliado A, Kaprio J, Koskenvuo M, Pukkala E, Skytthe A and Hemminki K (2000) Environmental and heritable factors in the causation of cancer. Analyses of cohorts of twins from Sweden, Denmark and Finland. *New Engl J Med* **343**: 78–85

Morita M, Kuwano H, Ohno S, Sugimachi K, Seo Y, Tomoda H, Furusawa M and Nakashima T (1994) Multiple occurence of carcinoma in the upper aerodigestive tract associated with esophageal cancer: reference to smoking, drinking and family history. *Int J Cancer* **58**: 207–210

Parshad R, Price FM, Bohr VA, Cowans KH, Zujewski JA and Sanford KK (1996) Deficient DNA repair capacity, a predisposing factor in breast cancer. *Br J Cancer* **74**: 1–5

Patel RK, Trevedi AH, Arora DC, Bhatavdekar JM and Patel DD (1997) DNA repair proficiency in breast cancer patients and their first-degree relatives. *Int J Cancer* **73**: 20–24

Peto J and Mack TM (2000) High constant incidence in twins and other relatives of women with breast cancer. *Nature Genet* **26**: 411–414

Rached E, Schindler R, Beer KT, Vetterli D and Greiner (1998) No predictive value of the micronucleus assay for patients with severe acute reaction of normal tissue after radiotherapy. *Eur J Cancer* **34**: 378–383

Roberts SA, Spreadborough AR, Bulman B, Barber JBP, Evans DGR and Scott D (1999) Heritability of cellular radiosensitivity: a marker of low penetrance predisposition genes in breast cancer? *Am J Human Genet* **65**: 784–794

Scott D, Spreadborough A, Levine E and Roberts SA (1994) Genetic predisposition to breast cancer. *Lancet* **344**: 1444

Scott D, Barber JB, Levine EL, Burrill W and Roberts SA (1998) Radiation-induced micronucleus induction in lymphocytes identifies a high frequency of radiosensitive cases among breast cancer cases: a test for predisposition? *Br J Cancer* **77**: 614–620

Scott D, Barber JBP, Spreadborough AR, Burrill W and Roberts SA (1999) Increased chromosomal radiosensitivity in breast cancer patients: a comparison of two assays. *Int J Radiat Biol* **75**: 1–10

Scott D, Roberts SA, Spreadborough, Bulman B, Barber JBP and Evans DGR (2000) Chromosomal radiosensitivity and cancer predisposition. In: *Radiation Research, Vol. 2, Congress Proceedings*. Moriarty M, Mothershill C, Seymour C, Edington M, Ward JF and Fry RJM (eds) (11th International Congress of Radiation Research), pp 470–471. Allen Press: Lawrence

Sellers TA (1996) Familial predisposition to lung cancer. In: *Genetic Predisposition to Cancer*, Eeles RA, Ponder BAJ, Easton DF and Horwich A (eds) pp 344–353. Chapman & Hall: London

Son YH and Kapp SD (1985) Oral cavity and oropharyngeal cancer in a younger population. *Cancer* **55**: 441–444

Teare MD, Wallace SA, Haris M, Howell A and Birch JM (1994) Cancer experience in the relatives of an unselected series of breast cancer patients. *Br J Cancer* **10**: 102–111

Terzoudi GI, Jung T, Hain J, Vrouvas J, Margaritis K, Donta-Bakoyiannis C, Makropoulos V, Angelakis PH and Pantelias GE (2000) Increased chromosomal radiosensitivity in cancer patients: the role of cdk1/cyclin-B activity level in the mechanisms involved. *Int J Radiat Biol* **76**: 607–615

Von Doersten PG, Cruz RM, Rasgon BM, Queensberry CP and Hilsinger RL (1995) Relation between age and head and neck cancer recurrence after surgery: a multivariate analysis. *Otolaryngol Head Neck* **113**: 197–203

© 2001 Cancer Research Campaign
